# Socio-ecological correlates of exercise procrastination and exercise addiction: a preliminary exploratory study using a single-university sample

**DOI:** 10.3389/fpsyt.2026.1787438

**Published:** 2026-03-25

**Authors:** Yuxia Wang, Hengzhi Deng, Xing Zhang, Hansen Li

**Affiliations:** 1 Department of Physical Education, Henan Logistics Vocational College, Zhengzhou, China; 2 Faculty of Sports and Exercise Science, University of Malaya, Kuala Lumpur, Malaysia; 3 Department of Physical Education and Sport, Faculty of Sport Sciences, University of Granada, Granada, Spain; 4 School of Physical Education, Sichuan Agricultural University, Yaan, China

**Keywords:** active lifestyle, exercise, mental health, physical activity, training

## Abstract

Exercise procrastination and exercise addiction reflect two maladaptive patterns at opposite ends of the physical-activity spectrum, yet their socio-ecological correlates among university students remain unclear. We surveyed 570 Chinese university students (mean age = 19.15 ± 1.09 years) and assessed exercise procrastination using the Procrastination in Exercise Scale (PES) and exercise addiction using the Revised Exercise Addiction Inventory (EAI-R). Candidate predictors were drawn from individual characteristics, health behaviors, and interpersonal network factors. To accommodate a relatively large predictor set, we applied LASSO regression with 10-fold cross-validation (λ_min) and evaluated robustness using 1,000 bootstrap resamples and 1,000 repetitions of random 10-fold cross-validation, emphasizing predictors retained in the primary models and consistently selected across both sensitivity analyses. For exercise addiction, the most robust predictors were regular exercise (positively associated) and female (vs. male) sex (negatively associated). For exercise procrastination, later habitual bedtime and more frequent short-video use before sleep were positively associated, whereas regular exercise and a greater number of friends were negatively associated. Regular exercise was the only shared correlate, but it showed opposite directions across outcomes—lower procrastination yet higher addiction-like risk. These findings indicate that campus health promotion may benefit from stratified strategies that both reduce exercise delay and monitor potentially excessive, addiction-like exercise tendencies. Limitations include the cross-sectional, self-reported, single-site design and a coarse personality measure, which restrict causal inference and generalizability; longitudinal replication with improved measurement is warranted.

## 1 Introduction

Physical activity is well established as beneficial for physical and mental health, lowering risks of major chronic diseases, mood disorders, and premature mortality (1, 2). Yet around one-third of adults worldwide still do not meet WHO recommendations, making inactivity a major modifiable risk factor that may be reinforced by urbanization-related lifestyle changes (3–5).

Increasing population-level physical activity is therefore a priority, but “more” is not always “better.” In some highly active individuals, excessive exercise can contribute to physiological strain, injuries, and psychological imbalance, and may develop into compulsive, addiction-like patterns. Although exercise addiction is less common than inactivity (≈3% in the general population, higher in exercise-engaged groups), its potential harms warrant attention (6–8).

Two constructs help describe extremes across the activity spectrum: exercise procrastination and exercise addiction. Exercise procrastination refers to repeated, irrational delay or avoidance of planned exercise despite knowing the negative consequences (9) and is linked to lower physical activity (10–12). Exercise addiction involves excessive, poorly controlled training with adverse physiological, psychological, and/or social outcomes (7), often co-occurring with injuries, emotional problems, and mental disorders (13–15)(Landolfi, 2013). Understanding the mechanisms behind “too little” and “too much” activity is thus important for public health.

Chinese university students are a key group for studying these patterns. University life increases discretionary time and opportunities to form exercise habits, while ubiquitous smartphone and social media use may foster problematic use and behavioral polarization, potentially amplifying both exercise procrastination and compulsive exercise (16, 17).

From a socio-ecological perspective, these behaviors reflect multilevel influences beyond willpower, including individual characteristics and interpersonal/family contexts (18, 19). While correlates of physical activity in university students have been examined, evidence remains limited for socio-ecological profiles of exercise procrastination and exercise addiction in Chinese students (20, 21). Therefore, this study applied a socio-ecological framework to identify individual, behavioral, and social-environmental characteristics associated with exercise procrastination and exercise addiction among Chinese university students, providing a basis for targeted interventions and future mechanism research.

## 2 Methods

### 2.1 Participants

This study recruited university students in public campus areas over a two-week period starting 1 November 2024. Research staff approached students and guided them to complete the smartphone-based questionnaire on site. Inclusion criteria were being a registered university student and physically able to participate in standard physical education courses. Exclusion criteria were abnormally fast completion, incomplete responses, and extreme values in open-ended numeric items (identified using the interquartile range method). Participants were informed about the study purpose and data use, and provided written informed consent. In total, 570 students completed the survey. The study was approved by the Ethics Committee of College of Physical Education at Southwest University.

### 2.2 Variables and measurements

Referring to the socio-ecological model and relevant empirical studies (22–25), this study selected independent variables from three dimensions: individual characteristics, health behaviors, and interpersonal network.

The purpose of using this framework was to include, at the initial variable-selection stage, variables that cover as many relevant domains as possible. Accordingly, our included variables comprised both commonly used, conservative sociodemographic indicators and several popular or emerging factors (e.g., screen time and bedtime phone use). This approach enabled us to balance tradition (i.e., facilitating comparisons with other studies using sociodemographic designs) and flexibility (i.e., incorporating some cutting-edge or currently salient factors). At the same time, we emphasize that questionnaires are generally not recommended to be excessively long (may reduce response rate) (26, 27); therefore, we could only consider and include a limited number of variables and, where possible, adopted brief measures to reduce respondents’ cognitive burden and time costs. However, it should be noted that this strategy inevitably introduces some measurement imprecision and conceptual ambiguity.

Specifically, we first drew on the existing literature on “physical activity and sociodemographic factors” to include a set of potential predictors at the individual and behavioral levels (e.g., age, sex, or marital status) (28, 29). We then, through discussions within the author team, specified a broader range of predictors, including factors at the community and policy levels. All variables were measured by self-report.

#### 2.2.1 Independent variables

##### 2.2.1.1 Individual characteristics

• Age: Recorded as self-reported integer age (years).
• Biological sex: Male = 0, Female = 1.
• Ethnicity: Han = 0, ethnic minority = 1.
• Grade level: 1 = first year, 2 = second year, 3 = third year, 4 = fourth year, 5 = postgraduate.
• Major: 0 = natural sciences, 1 = humanities and social sciences.
• Monthly household income:
1 = 0–5,000 RMB,
2 = 5,001–10,000 RMB,
3 = 10,001–15,000 RMB,
4 = 15,001–20,000 RMB,
5 = 20,001–25,000 RMB,
6 = 25,001–30,000 RMB,
7 = >30,000 RMB.
• Personality type (MBTI):
1 = unknown/not assessed,
2 = introverted personality,
3 = extroverted personality.

It should be noted that we included MBTI results because this standardized test has become highly popular among Chinese university students in recent years. Because the full MBTI questionnaire is quite lengthy, we were unable to administer it within this survey (as it would have imposed excessive time burden and cognitive load). Instead, we asked participants to report their existing MBTI results. Accordingly, the “Unknown” category does not represent missing data; rather, it indicates participants who have not taken the MBTI test (2).

##### 2.2.1.2 Individual behaviors

• Smoking: 1 = non-smoker, 2 = smoker.
• Regular exercise (i.e., whether the participant engages in physical activity on a fixed schedule or at regular intervals, reflecting the temporal stability of the behavior): 0 = no, 1 = yes.
• Use of nutritional supplements: 0 = no, 1 = yes.
• Habitual bedtime at night:
1 = before 20:00,
2 = 20:00–21:59,
3 = 22:00–23:59,
4 = 00:00–01:59,
5 = after 02:00.
• Short-video use before sleep.
1 = never,
2 = sometimes,
3 = often,
4 = always.
• Typical nighttime sleep duration: Recorded as self-reported hours.
• Average daily screen time (mobile phone, computer, etc.): Recorded as self-reported hours.

##### 2.2.1.3 Interpersonal network

• Living arrangement:
1 = living alone,
2 = living with parents,
3 = living with classmates or friends,
4 = living with spouse/partner.
• Relationship status:
1 = single,
2 = in a romantic relationship,
3 = married,
4 = other.
• Household size: Recorded as self-reported number of family members.
• Number of friends often engaging in leisure activities together in the past month: Recorded as self-reported number.

#### 2.2.2 Dependent variables

##### 2.2.2.1 Exercise procrastination

Exercise procrastination was assessed using the six-item Procrastination in Exercise Scale (9), rated on a 5-point Likert scale (1–5), with higher scores indicating greater procrastination. We translated the PES into Chinese following established cross-cultural procedures (30). Internal consistency in this study was high (Cronbach’s α = 0.90).

##### 2.2.2.2 Exercise addiction

Exercise addiction was assessed using the Revised Exercise Addiction Inventory (31), a six-item self-report scale rated on a 6-point Likert scale (1–6), with higher scores indicating greater addiction tendency. We used the Chinese version, which retains five items and shows a one-factor structure (32). Internal consistency in this study was acceptable (Cronbach’s α = 0.80).

### 2.3 Statistical analysis

#### 2.3.1 Primary analysis: LASSO variable selection

Continuous variables (e.g., age) and ordinal categories (e.g., education, income) were treated as continuous; binary variables (e.g., sex) were entered directly, and multi-category nominal variables (e.g., smoking, personality, living arrangement, relationship status) were modeled as factors. Given the relatively large predictor set and limited sample size, we adopted Least Absolute Shrinkage and Selection Operator (LASSO) approaches used in prior studies (33–35).

We applied LASSO regression for simultaneous variable selection and regularization by shrinking some coefficients to zero via a penalty term (λ), yielding a parsimonious model (36). Compared with stepwise regression, LASSO is generally more robust and accurate (24, 37, 38).

Because multicollinearity can affect LASSO (39), we first checked variance inflation factors (VIF), using VIF < 5 as the threshold; no multicollinearity was detected (40). LASSO models were fit using glmnet (alpha = 1) with 10-fold cross-validation to select λ, and predictors with non-zero coefficients in the final model were retained (41). We also report the coefficient path estimated on the full dataset to show shrinkage across λ values.

#### 2.3.2 Sensitivity analysis 1: bootstrap stability of variable selection

To assess the stability of LASSO selection, we performed 1,000 bootstrap resamples with replacement (42–44). In each resample, λ was re-tuned via 10-fold cross-validation using the same settings. We then summarized each predictor’s selection frequency (non-zero coefficient proportion) and sign consistency (proportion of positive vs. negative signs when selected). Predictors with selection frequency ≥ 0.90 and sign consistency ≥ 0.90 were flagged as stable signals (43, 45–47). Median penalized coefficients across resamples were also reported as descriptive, shrunken effect indicators.

#### 2.3.3 Sensitivity analysis 2: repeated random 10-fold cross-validation

As a complementary check for sensitivity to fold assignment, we repeated 10-fold cross-validation 1,000 times with different random fold splits. In each run, LASSO was tuned with the same settings; we then computed selection frequency and sign consistency across repetitions as in the bootstrap analysis. All resampling and fold generation used a fixed random seed (set.seed (20251115)) to ensure reproducibility. All analyses were conducted in R (version 4.5.1) using the glmnet package.

## 3 Results

### 3.1 Participant characteristics

A total of 570 students were included (mean age = 19.15 ± 1.09 years; median = 19, IQR = 18–20) (**Supplementary Table 1**). The sample comprised 298 participants in sex category 1 (52.3%) and 272 in category 2 (47.7%). Most were Han ethnicity (96.3%). Grade distribution was Year 1 (58.2%), Year 2 (22.8%), Year 3 (15.3%), and Year 4 (3.7%). Majors were evenly split between natural sciences (49.1%) and humanities/social sciences (50.9%). For household income, 41.9% reported 0–5,000 RMB and 33.3% reported 5,001–10,000 RMB/month. Most participants were non-smokers (87.4%); 48.8% reported regular exercise and 16.7% reported nutritional supplement use. Bedtime was mainly 22:00–23:59 (53.5%) or 00:00–01:59 (38.1%). Short-video use before sleep was frequent (often: 38.2%; always: 25.1%). Median nighttime sleep duration was 7 h (IQR = 7–8) and daily screen time was 7 h (IQR = 5–9). Median household size was 4 (IQR = 4–5) and the median number of close leisure friends was 5 (IQR = 3–6). Most students lived with classmates/friends (73.3%) and were single (72.3%).

### 3.2 Primary results (LASSO)

**Figure 1:**
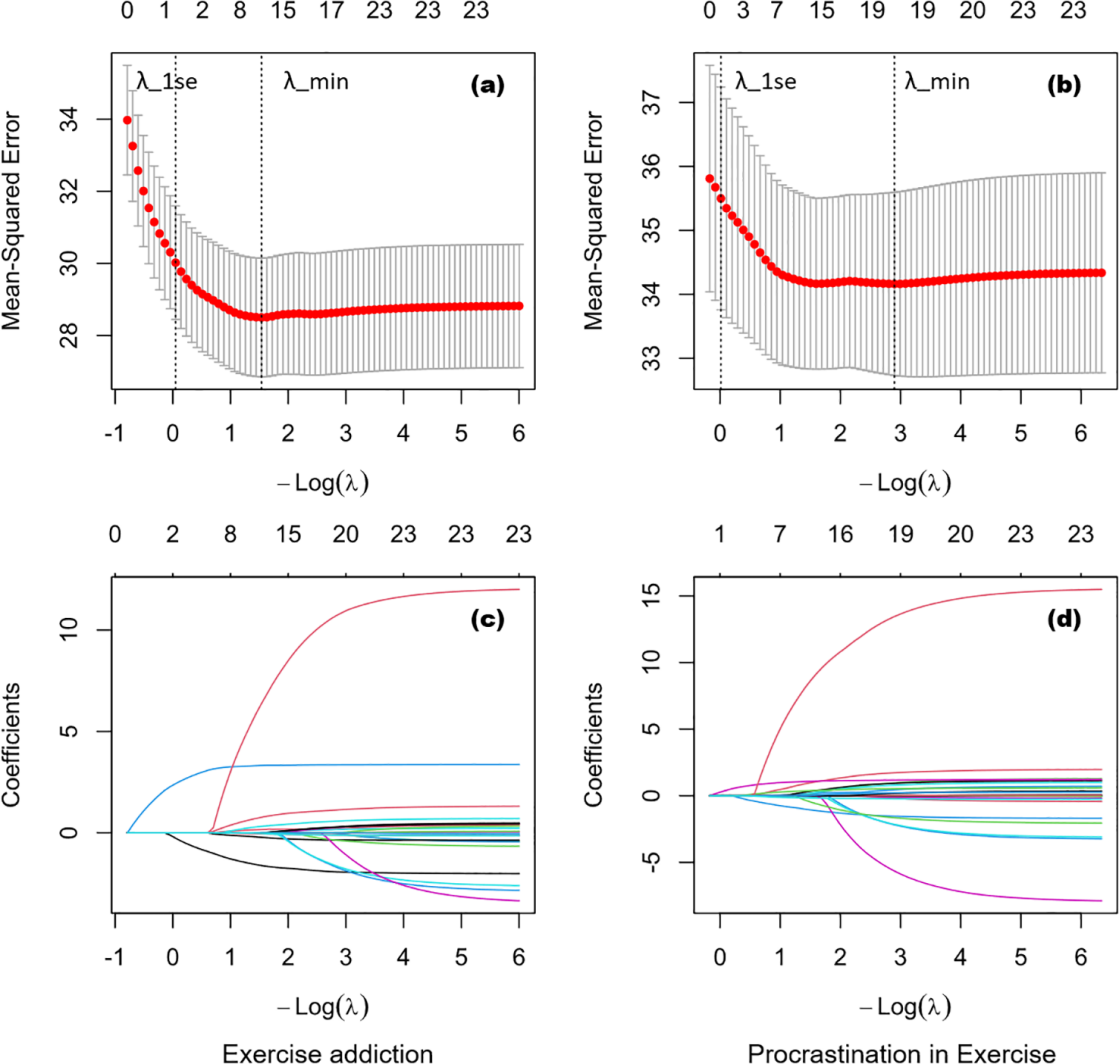
LASSO regression: cross-validated prediction error and coefficient shrinkage paths. **(a, b)** show the relationship between the cross-validated mean squared error (MSE) and the penalty parameter (−log(λ)). The bottom x-axis indicates the corresponding −log(λ) values, and the top x-axis indicates the number of predictors with non-zero coefficients at each penalty level. **(c, d)** display the coefficient paths fitted on the full sample (without cross-validation), illustrating how predictor coefficients are progressively shrunk toward zero as the penalty increases.

**Figure 1** presents the 10-fold cross-validation (CV) curves and coefficient paths for the LASSO models predicting exercise addiction (EAI-R score) and exercise procrastination (PES score). Panels (a)–(b) show the cross-validated prediction error (mean squared error) as a function of the penalty parameter (-log (λ)), with the two dashed vertical lines indicating λ_min (the λ yielding the minimum mean CV error) and λ_1se (the largest λ within one standard error of the minimum). In our data, λ_1se produced an overly sparse solution for exercise addiction and procrastination (as shown in **Figures 1a, b**). Since our primary goal was to identify potentially important correlates rather than to build the most parsimonious predictive model. Therefore, we adopted λ_min for estimation (exercise addiction: λ_min = 0.215; exercise procrastination: λ_min = 0.055).

In the primary LASSO models (**Table 1**), exercise addiction retained nine predictors with non-zero coefficients, including relationship status (married vs single), regular exercise, sex (female vs male), personality contrasts, major, age, screen time, and number of friends. Exercise procrastination retained a broader set of predictors spanning relationship status, living arrangement, personality, regular exercise, bedtime, and several behavioral and social indicators (**Table 1**).

**Table 1:** LASSO-selected predictors (non-zero penalized coefficients).

Predictor (interpretable label)	Exercise addiction coef.	Exercise procrastination coef.
Relationship status (married vs single)	6.378	13.453
Relationship status (other vs single)	—	-1.657
Relationship status (in a romantic relationship vs single)	—	0.178
Living arrangement (with spouse/partner vs living alone)	—	-5.654
Living arrangement (with parents vs living alone)	—	-2.183
Living arrangement (with classmates/friends vs living alone)	—	-2.150
Regular exercise (yes vs no)	3.330	-1.539
Sex (female vs male)	-1.611	—
Age (years)	0.168	-0.190
Major (humanities & social sciences vs natural sciences)	0.327	—
Personality (extroverted vs unknown/not assessed)	0.770	1.721
Personality (introverted vs unknown/not assessed)	-0.217	0.934
Screen time (hours/day)	-0.047	0.021
Number of friends (past month; count)	-0.028	-0.236
Habitual bedtime (later category)	—	1.174
Ethnicity (ethnic minority vs Han)	—	0.964
Grade level (higher year)	—	0.451
Nutritional supplement use (yes vs no)	—	0.786
Short-video use before sleep (higher frequency)	—	0.528
Household size (persons)	—	0.218
Monthly household income (higher category)	—	-0.099

### 3.3 Bootstrap stability

Applying a stringent stability criterion (selection frequency ≥ 0.90 in bootstrap and ≥ 0.90 in repeated CV), exercise addiction showed three highly stable predictors (**Supplementary Table 2A**): regular exercise (positive), sex (female vs male; negative), and extroverted personality (vs unknown; positive). All three displayed highly consistent directions across resampling, with regular exercise and sex selected in 100% of bootstrap samples.

For exercise procrastination, five predictors met the same high-stability criterion (**Supplementary Table 2B**): later bedtime (positive), regular exercise (negative), more friends (negative), extroverted personality (positive), and more frequent short-video use before sleep (positive), each showing strong directional consistency and being selected in ≥92.5% of bootstrap samples.

### 3.4 Repeated random 10-fold CV

In the repeated random 10-fold CV analysis (**Supplementary Table 3**), exercise addiction showed eight highly stable predictors (select_freq ≥ 0.90), including sex (negative), age (positive), major (positive), personality contrasts (introverted negative; extroverted positive), regular exercise (positive), relationship status (married vs single; positive), and screen time (negative). For exercise procrastination, eight predictors met the same ≥0.90 criterion, including grade level, extroverted personality, regular exercise (negative), bedtime, short-video use before sleep, relationship status (married vs single; positive), number of friends (negative), and introverted personality (positive).

## 4 Discussion

### 4.1 Summary of findings

In the present study, we used a socio-ecological framework and a LASSO-based selection approach, complemented by two robustness checks, to identify correlates of exercise procrastination and exercise addiction among Chinese university students.

To enhance the robustness of our findings, we adopted a conservative criterion for interpretation: we only retained predictors that remained statistically supported in the primary model and were consistently replicated across all prespecified sensitivity analyses, whereas predictors that appeared significant only in the primary analysis or only in some sensitivity specifications were treated as unstable and were not emphasized in the interpretation.

Under this criterion, a compact set of stable predictors emerged. For exercise addiction, three correlates showed the most robust signals: regular exercise was positively associated with addiction scores, female (vs. male) sex was negatively associated, and extroverted personality (vs. unknown/not assessed) was positively associated.

For exercise procrastination, five predictors consistently survived all analyses: later habitual bedtime and more frequent short-video use before sleep were positively associated with exercise procrastination, whereas regular exercise and a greater number of friends were negatively associated; extroverted personality (vs. unknown/not assessed) again showed a positive association. Notably, regular exercise was the only predictor shared by both outcomes, but it displayed opposite directions (higher addiction risk yet lower procrastination).

It is important to emphasize that the present study is based on cross-sectional data. Accordingly, all results should be interpreted as associations observed at a single time point, rather than evidence of temporal ordering, directional effects, or causal mechanisms. Throughout the discussion, any theoretical interpretations are offered strictly as hypothesis-generating explanations. Reverse causation and unmeasured confounding remain plausible.

Taken together, the findings suggest that campus health promotion should attend to risks at both ends of the activity spectrum and develop stratified, tailored intervention strategies based on distinct risk profiles.

### 4.2 Comparison with previous studies

#### 4.2.1 Exercise addiction

Regarding exercise addiction, we found that regular exercise was significantly and positively associated with exercise addiction scores. This result is broadly consistent with prior literature, suggesting that the risk of exercise addiction is concentrated mainly among individuals who exercise frequently, train with higher volumes, and have higher training levels (e.g., athletes) (48–50). For people who exercise regularly, exercise may be an important means of maintaining an ideal physique, engaging in social interaction, and reducing negative emotions. As a result, they may obtain greater “rewards” from exercise, which could make them more prone to developing addictive tendencies.

In terms of personality, although we observed a difference between students reporting an introverted personality and those classified as “unknown,” the “unknown” category does not reflect a distinct personality trait or a meaningful subgroup. Rather, it indicates participants who had not previously taken an MBTI assessment and therefore could not report a result. Accordingly, this contrast should be interpreted with caution, cannot be meaningfully attributed to personality differences, and should not be used to support any substantive or generalizable conclusions.

Regarding sex differences, we observed that being female was associated with lower levels of exercise addiction. This aligns with previous findings suggesting that men are more likely to develop exercise addiction (51). Prior research has shown that male athletes tend to demonstrate greater investment in training and higher “vigor” in affective terms (52). This pattern may be related to behavioral differences driven by sex hormones (53).

#### 4.2.2 Exercise procrastination

Regarding exercise procrastination, we found that later bedtimes and watching short videos before sleep were associated with more severe exercise procrastination. Previous studies have shown that using mobile phones before sleep is related to bedtime procrastination, which in turn may lead to poorer sleep quality (54–56). In addition, later sleep timing is also associated with poorer sleep quality (57). Furthermore, poorer sleep quality may contribute to procrastination, as supported by research on workplace procrastination (58). This may be explained by reduced self-control resources resulting from inadequate sleep (59).

In terms of regular exercise, we found that individuals who exercise regularly were less likely to procrastinate on exercise. This result is unsurprising. During the scale development stage of the tool we used, Kelly and Walton (9) observed that exercise procrastination negatively predicted physical activity levels. Based on cross-sectional mediation analyses, others have argued that procrastination may reduce exercise behavior among university students by lowering exercise commitment and action control (60). This is consistent with our observations. One difference is that we treated regular exercise as a predictor rather than an outcome, because we assumed that individuals with stable behavioral routines are less likely to develop procrastination, including exercise procrastination. Zhuan, Cao (11) adopted a similar view, proposing that engaging in physical exercise helps maintain physical health and strengthens one’s courage to face delayed tasks, thereby serving as an effective strategy to reduce procrastination. Supporting evidence also exists: cross-lagged models indicate that exercise intention predicts subsequent exercise procrastination, suggesting that individuals with stronger exercise intentions may be less likely to show exercise procrastination (10). That study also suggested that the temporal relationship between exercise procrastination and physical activity levels may be bidirectional (10), highlighting the complexity of their developmental dynamics. Despite differences in data and analytic assumptions, our study and the studies above jointly support an association between exercise procrastination and exercise/physical activity.

In addition, we observed that having more friends was associated with lower exercise procrastination. First, a larger friendship network may provide stronger social support and companionship (easier to find someone to go with, easier to make plans), shifting the “startup cost” of beginning exercise from individual willpower to a shared activity that is easier to carry out. Second, having more friends increases the likelihood of being exposed to contexts in which “people around me are exercising,” which may activate social norms and peer modeling effects, increase the priority of exercise and self-efficacy, and reduce the psychological space for “I’ll do it later.” Third, friendships may create mild supervision and commitment pressure (it is harder to cancel after making plans), while also buffering stress and improving mood through interaction and emotional support, making exercise more likely to be used as a positive daily regulation strategy.

Finally, similar to the exercise addiction results, although we observed a predictive effect of personality, the comparison category was extroverted personality vs. unknown/not assessed. This result is not generalizable; therefore, we do not provide further interpretation.

### 4.3 Limitations

Several limitations should be considered when interpreting the present findings.

This study did not assess exercise-related online interactions or tool-use characteristics (e.g., whether fitness apps were used for workout guidance vs. social comparison, participation in online challenges or virtual accountability groups, and engagement with sports-related content across platforms). As a result, we could not evaluate whether different digitally mediated exercise-promotion pathways have distinct implications—community support and accountability may be linked to lower procrastination, whereas gamification and social comparison (e.g., step counts, workout streaks, leaderboards) may relate to more compulsive participation and higher addiction risk. Future studies should use more fine-grained items or validated scales to differentiate these online usage patterns.

Limitations also remain at the psychological layer of the socio-ecological model. Although post-pandemic anxiety and stress are plausible drivers of both procrastination and addiction (61, 62), we did not measure these emotions; therefore, we could not test mediation (or moderated mediation) pathways linking key predictors to outcomes. Given the exploratory aim of identifying potential correlates, future work should incorporate brief validated measures (e.g., GAD-7, PSS-10) and use longitudinal or experimental designs to clarify directionality and mechanisms.

Our sample was drawn from a single university, with an imbalanced grade distribution (58.2% first-year students), which limits generalizability across diverse higher-education contexts in China. Institutional differences in PE requirements, facility access, campus exercise culture, and peer-network structures may modify observed associations. Future research should adopt multi-center, stratified sampling and include school-level indicators (e.g., facility provision, mandatory PE policies) to enable subgroup or multilevel analyses.

Finally, “regular exercise” was measured as a binary (yes/no) indicator intended to capture habit stability rather than activity volume. This coarse measure cannot differentiate modality, dose, or goals (e.g., team vs. individual sports; aerobic vs. resistance; intensity/frequency; health vs. appearance/competition), which may attenuate or mask heterogeneous associations with procrastination and addiction. Future studies should retain a habit-based indicator while adding more granular assessment (e.g., IPAQ-SF plus modality/goal items) and testing interactions in larger samples.

## 5 Conclusion

Using a socio-ecological framework and a LASSO-based selection strategy with two robustness checks (bootstrap resampling and repeated random 10-fold cross-validation), this study identified a small set of stable correlates of exercise procrastination and exercise addiction among Chinese university students. For exercise addiction, two substantial predictors consistently survived all analyses: regular exercise was positively associated with addiction scores, female (vs. male) sex was negatively associated. For exercise procrastination, four robust predictors emerged: later habitual bedtime and more frequent short-video use before sleep were positively associated with procrastination, whereas regular exercise and a larger number of friends were negatively associated. Overall, these findings underscore the need for campus health promotion to address risks at both ends of the activity spectrum. Future research should replicate these correlates in more diverse samples and test their temporal and causal relevance using longitudinal designs or targeted interventions with improved measurement precision.

## Data Availability

The raw data supporting the conclusions of this article will be made available by the authors, without undue reservation.
