# Supplementary material for: Socio-ecological correlates of exercise procrastination and exercise addiction: a preliminary exploratory study using a single-university sample
Source: Front Psychiatry. 2026 Mar 25;17:1787438. doi: 10.3389/fpsyt.2026.1787438 (PMC13056851; doi:10.3389/fpsyt.2026.1787438)
Supplement: Supplementary file 1 [file Table1.docx]

Supplementary Material

**Table S1. Participant characteristics**

| **Variable** | **Category / unit** | **n (%)** | **Mean (SD)** | **Median (IQR)** |
| --- | --- | --- | --- | --- |
| Sex | Male | 298 (52.3) |  |  |
|  | Female | 272 (47.7) |  |  |
| Ethnicity | Han | 549 (96.3) |  |  |
|  | Ethnic minority | 21 (3.7) |  |  |
| Grade level | Year 1 | 332 (58.2) |  |  |
|  | Year 2 | 130 (22.8) |  |  |
|  | Year 3 | 87 (15.3) |  |  |
|  | Year 4 | 21 (3.7) |  |  |
| Major | Natural sciences | 280 (49.1) |  |  |
|  | Humanities & social sciences | 290 (50.9) |  |  |
| Monthly household income (RMB) | 0–5,000 | 239 (41.9) |  |  |
|  | 5,001–10,000 | 190 (33.3) |  |  |
|  | 10,001–15,000 | 73 (12.8) |  |  |
|  | 15,001–20,000 | 35 (6.1) |  |  |
|  | 20,001–25,000 | 18 (3.2) |  |  |
|  | 25,001–30,000 | 1 (0.2) |  |  |
|  | >30,000 | 14 (2.5) |  |  |
| Personality type (MBTI item) | Unknown / not assessed | 223 (39.1) |  |  |
|  | Introverted | 219 (38.4) |  |  |
|  | Extroverted | 128 (22.5) |  |  |
| Smoking | Non-smoker | 498 (87.4) |  |  |
|  | Traditional cigarettes | 72 (12.7) |  |  |
|  | E-cigarettes | 2 (0.4) |  |  |
| Regular exercise | No | 292 (51.2) |  |  |
|  | Yes | 278 (48.8) |  |  |
| Nutritional supplement use | No | 475 (83.3) |  |  |
|  | Yes | 95 (16.7) |  |  |
| Habitual bedtime | Before 20:00 | 15 (2.6) |  |  |
|  | 20:00–21:59 | 25 (4.4) |  |  |
|  | 22:00–23:59 | 305 (53.5) |  |  |
|  | 00:00–01:59 | 217 (38.1) |  |  |
|  | After 02:00 | 8 (1.4) |  |  |
| Living arrangement | Living alone | 31 (5.4) |  |  |
|  | Living with parents | 120 (21.1) |  |  |
|  | Living with classmates/friends | 418 (73.3) |  |  |
|  | Living with spouse/partner | 1 (0.2) |  |  |
| Relationship status | Single | 412 (72.3) |  |  |
|  | In a romantic relationship | 145 (25.4) |  |  |
|  | Married | 1 (0.2) |  |  |
|  | Other | 12 (2.1) |  |  |
| Short-video use before sleep | Never | 45 (7.9) |  |  |
|  | Sometimes | 164 (28.8) |  |  |
|  | Often | 218 (38.2) |  |  |
|  | Always | 143 (25.1) |  |  |
| Age (years) | — | — | 19.15 (1.09) | 19 (18–20) |
| Nighttime sleep duration (hours) | — | — | 7.36 (0.77) | 7 (7–8) |
| Daily screen time (hours) | — | — | 6.81 (3.04) | 7 (5–9) |
| Household size (persons) | — | — | 4.33 (0.94) | 4 (4–5) |
| Number of friends (past month) | — | — | 4.74 (2.45) | 5 (3–6) |

**Table S2. Sensitivity analysis: Bootstrap stability**

**A) Exercise addiction**

| **Predictor (interpretable label)** | **Freq** | **Direction (pos/neg)** | **Median penalized coef.** |
| --- | --- | --- | --- |
| Regular exercise (Yes vs no) | 1.000 | Pos (1.000 / 0.000) | 3.295 |
| Sex (Female vs male) | 1.000 | Neg (0.000 / 1.000) | -1.732 |
| Personality (Extroverted vs unknown/not assessed) | 0.902 | Pos (0.997 / 0.003) | 0.934 |

**B) Exercise procrastination**

| **Predictor (interpretable label)** | **Freq** | **Direction (pos/neg)** | **Median penalized coef.** |
| --- | --- | --- | --- |
| Habitual bedtime (later category) | 0.997 | Pos (0.999 / 0.001) | 1.112 |
| Regular exercise (Yes vs no) | 0.993 | Neg (0.001 / 0.999) | -1.403 |
| Number of friends (count) | 0.985 | Neg (0.001 / 0.999) | -0.224 |
| Personality (Extroverted vs unknown/not assessed) | 0.960 | Pos (0.999 / 0.001) | 1.505 |
| Short-video use before sleep (higher frequency) | 0.925 | Pos (0.991 / 0.009) | 0.481 |

**Note.** Only predictors with bootstrap selection frequency ≥ 0.90 are shown; Pos, positive; Neg, Negative

**Table S3. Sensitivity analysis: Repeated random 10-fold CV stability**

**3A) Exercise addiction**

| **Predictor** | **Select freq** | **Direction** |
| --- | --- | --- |
| Sex (Female vs male) | 1.000 | Negative |
| Age (years) | 1.000 | Positive |
| Major (Humanities/social sciences vs natural sciences) | 1.000 | Positive |
| Personality (Introverted vs unknown) | 1.000 | Negative |
| Personality (Extroverted vs unknown) | 1.000 | Positive |
| Regular exercise (Yes vs no) | 1.000 | Positive |
| Relationship status (Married vs single) | 1.000 | Positive |
| Screen time (hours/day) | 0.999 | Negative |

**3B) Exercise procrastination**

| **Predictor** | **Select freq** | **Direction** |
| --- | --- | --- |
| Grade level (higher year) | 1.000 | Positive |
| Personality (Extroverted vs unknown) | 1.000 | Positive |
| Regular exercise (Yes vs no) | 1.000 | Negative |
| Habitual bedtime (later category) | 1.000 | Positive |
| Short-video use before sleep (higher frequency) | 1.000 | Positive |
| Relationship status (Married vs single) | 1.000 | Positive |
| Number of friends (count) | 1.000 | Negative |
| Personality (Introverted vs unknown) | 0.952 | Positive |

*Note. Only predictors with select_freq ≥ 0.90 are shown.*
